# Transanal minimally invasive surgery: impact on quality of life and functional outcome

**DOI:** 10.1007/s00464-015-4326-3

**Published:** 2015-07-03

**Authors:** Maria Verseveld, Renée M. Barendse, Martijn P. Gosselink, Cornelis Verhoef, Eelco J. R. de Graaf, Pascal G. Doornebosch

**Affiliations:** Department of General Surgery, IJsselland Hospital, P.O. Box 960, Capelle aan den Ijssel, The Netherlands; Department of Surgery, Academic Medical Centre, Amsterdam, The Netherlands; Department of Surgery, Oxford University Hospital, Oxford, UK; Department of Surgery, Erasmus MC Cancer Institute, Rotterdam, The Netherlands

**Keywords:** Transanal minimally invasive surgery, Anorectal function, Transanal endoscopic microsurgery, Faecal incontinence, Quality of life

## Abstract

**Background:**

Transanal minimally invasive surgery (TAMIS) is emerging as an alternative to transanal endoscopic microsurgery. Quality of life (QOL) and functional outcome are important aspects when valuing a new technique. The aim of this prospective study was to assess both functional outcome and QOL after TAMIS.

**Methods:**

From 2011 to 2013, patients were prospectively studied prior to and at least 6 months after TAMIS for rectal adenomas and low-risk T1 carcinomas using a single-site laparoscopy port. Functional outcome was determined using the Faecal Incontinence Severity Index (FISI). Quality of life was measured using functional [Faecal Incontinence Quality of Life (FIQL)] and generic (EuroQol EQ-5D) questionnaires.

**Results:**

The study population consisted of 24 patients 13 men, median age 59 (range 42–83) with 24 tumours [median distance from the dentate line 8 cm (range 2–17 cm); median tumour size 6 cm^2^ (range 0.25–51 cm^2^); 20 adenomas; 4 low-risk T1 carcinomas]. Post-operative complications occurred in one patient (4 %; grade IIIb according to Clavien Dindo classification). Compared to baseline, FISI remained unaffected (9.8 vs 7.3; *P* = 0.26), FIQL remained unaffected, and EuroQol EQ-5D improved (EQ-VAS: 77 vs 83; *P* = 0.04).

**Conclusion:**

There was no detrimental effect of TAMIS on anorectal function. Overall QOL was improved after TAMIS, probably due to removal of the tumour, and at 6 months was equal to the general population.

## Background

For the local resection of rectal adenomas and selected rectal carcinomas, transanal endoscopic microsurgery (TEM), as described by Buess, has emerged as the treatment of choice as it is superior to other local excision techniques [1–3]. Earlier studies have already shown that TEM has no impact on anorectal function and improves quality of life (QOL) [4–7]. Nevertheless, TEM is not being broadly incorporated into the surgical armamentarium. This may be explained by its high costs and long learning curve [8, 9].

Since 2010, single-site surgical ports are used as an alternative to the classical TEM rectoscope in transanal surgery. To date, many types of single ports have been explored transanally, such as the single-incision laparoscopic surgery port (SILS, Covidien, Mansfield, MA), the Single Site Laparoscopic Access System (SSL, Ethicon Endo-Surgery, Cincinnati, OH) and the Gelpoint Path platform (Applied Medical, Rancho Santa Margarita, CA). Recently, the acronym TAMIS, meaning *transanal minimally invasive surgery*, is suggested to avoid commercial links. TAMIS seems to be embraced by colorectal surgeons more than TEM and has already proven to be a feasible and safe modification [10]. Furthermore, the technique of TAMIS is advocated to be easier to learn, and because no specialized insufflator or operating rectoscope is needed, it is more readily available.

As a next step, efficacy of TAMIS should be balanced against TEM, including its effect on anorectal function and QOL. To date, however, impact of TAMIS on the functional outcome and QOL is reported only scarcely and indirectly [11].

The aim of this prospective study was to analyse the functional outcome as well as QOL after TAMIS.

## Materials and methods

### Patients

The study population consisted of patients who were referred for local excision of a rectal tumour between May 2011 and April 2013. All patients were evaluated preoperatively according to a standard protocol including rigid rectoscopy, tumour biopsy and endorectal ultrasound. Only rectal adenomas and low-risk T1 carcinomas, i.e. well differentiated, no signs of lymphangio-invasion and <3 cm, were considered eligible for this study. Patients with a pre-existing stoma, patients who underwent conversion to another technique, patients in whom histology results post-operative revealed a >T1 carcinoma and patients who underwent a combined operation were excluded.

Institutional review board approval was given prior to the commencement of the study, and in all patients, written informed consent was obtained.

### Surgical procedure

Procedures were performed by two surgeons who are extensively (>500) experienced in TEM and moderately (50–100) experienced in TAMIS (P.D. and E.d.G.). TAMIS was performed using the Single Site Laparoscopic Access System (SSL, Ethicon Endo-Surgery, Cincinnati, OH), as previously described [10]. In brief, this procedure is performed by using a 360° rotatable port in combination with a 30° laparoscope, providing easy and quick reorientation of the instrumentation and easy specimen collection. A single enema was given 1 h before surgery. Preoperative antibiotics (cefazoline/metronidazole) were administered. All patients were operated under general anaesthesia in the lithotomy position. A pneumorectum of 12–15 mmHg was established using carbon dioxide insufflation. A full-thickness excision was performed. At the surgeon’s discretion, the rectal wall defect was closed using a self-anchoring continuous suture. Operative time was defined as the time of inserting the SSL retractor until removal.

### Data collection

An independent research coordinator not previously involved in the patients’ care collected all data. Demographics, operative details, post-operative length of stay, post-operative complications and functional outcome were recorded for each patient.

Before and 6 months after TAMIS, patients were asked to fill out a questionnaire to assess anorectal function and QOL. We evaluated functional outcome by means of a detailed questionnaire based on the Faecal Incontinence Severity Index (FISI) (range 0–61) [12]. Quality of life was evaluated using the EuroQol EQ-5D/EQ-VAS scores (both, range 0–100) and the Faecal Incontinence Quality of Life (FIQL) score [overall score and four domains (lifestyle issues, coping–behaviour, depression and self-perception and embarrassment) (all, range 1–4)]. [13] The EuroQol EQ-5D/VAS scores were compared with a sex- and age-matched, community-based sample of healthy persons without co-morbidity [14]. Data are presented as medians and ranges. Changes within groups were evaluated using the nonparametric one-sample Wilcoxon’s signed-rank test. Comparison of these changes between groups was conducted using the Mann–Whitney *U* test. A *P* value of ≤0.05 was considered statistically significant.

## Results

Between May 2011 and April 2013, 50 patients were found eligible for this study. Eighteen patients were excluded; in 14 patients, TAMIS was combined with another surgical technique and four patients required additional surgery because of high-risk T1 or more invasive carcinoma.

Of the remaining 32 patients, 24 completed both preoperative and post-operative questionnaires (response rate 75 %) and were included for analysis. All patients had a minimal follow-up of 6 months (range 6–8). Eight patients did not provide us both the completed preoperative and post-operative questionnaires despite their informed consent and frequent encouragements. None of the eight non-responding patients developed an early recurrence. Two patients experienced post-operative haemorrhage, both treated conservatively. Hence, the reason for non-responding is not quite clear.

The group consisted of 13 males and 11 females. Median age was 59 years (range 42–83). Median distance from the distal tumour margin to the dentate line was 8 cm (range 2–17 cm), and median tumour size was 6 cm^2^ (range 0.25–51 cm^2^). Twenty-four tumours were removed: 20 adenomas and 4 low-risk T1 carcinomas. The median proportion of the rectal circumference covered by the lesion was 25 % (range 5–50). Median operative time was 32 min (range 13–94). One patient (4 %) experienced a complication consisting of haemorrhage requiring re-operation (grade IIIb according to Clavien Dindo classification). In hospital, mortality rate was zero. Median length of stay was 1 day (range 1–3 days; Table 1).

**Table 1:** Procedure-related characteristics

Median duration of operation in minutes (range)	32 (13–94)
Complications (N)	1/24 (4.2 %)
Re-operation for re-bleeding	1
Median length of hospital stay in days (range)	1 (1–3)

The mean FISI score decreased from 9.8 ± 2.3 to 7.3 ± 2.2 (*P* = 0.26). Fifteen patients were completely continent after surgery (63 %). Five patients (21 %) had a minor deterioration in FISI score of 8 (range 5–12).

**Fig. 1:**
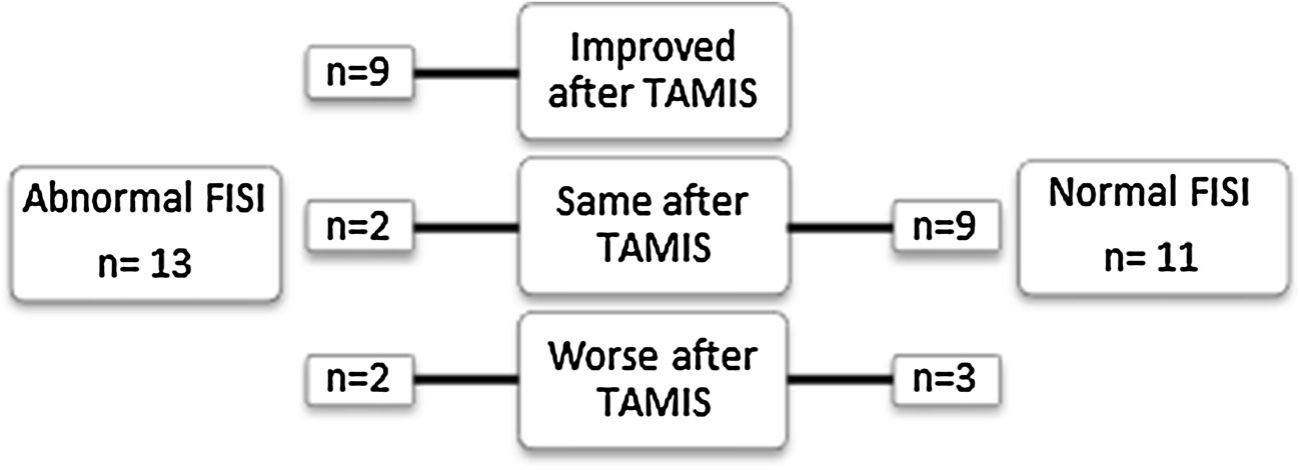
FISI-scores before and after transanal minimally invasive surgery (TAMIS)

The five patients who experienced an increase in FISI score had a significant shorter tumour distance to the dentate line (4.4 vs 7.4 cm; *P* = 0.04) and a significantly larger tumour size (21 vs 9 cm^2^; *P* = 0.05). The EQ-VAS score in these patients was significantly lower (71 vs 86; *P* = 0.03). A schematic overview is provided in Fig. 1.

The FIQL scores are shown in Table 2. A significant improvement in the FIQL subscale “coping behaviour” was seen post-operatively (*P* = 0.02). In patients in whom the FISI score deteriorated, the FIQL scores were lower at 6 months after TAMIS in all four dimensions (All *P* < 0.05). The size of the tumour and distance to the anal verge had no significant effect on these FIQL scores.

**Table 2:** Faecal Incontinence Quality of Life scores

	Preoperative	6 months after SPTS	*P* value
Lifestyle	3.8 (0.6)	3.9 (0.4)	0.15
Coping behaviour	3.0 (0.8)	3.6 (0.5)	0.02
Depression	3.6 (0.8)	3.7 (0.5)	0.27
Embarrassment	3.5 (0.5)	3.7 (0.4)	0.08
Total	3.5 (0.6)	3.7 (0.5)	0.12

The general QOL, as evaluated by EQ-VAS and EQ-5D, is presented in Table 3. From a patient perspective, the mean general QOL score (EQ-VAS) improved 6 months after TAMIS compared to baseline (*P* = 0.03). From a social perspective, the mean EQ-5D index score remained equal. EQ-VAS and EQ-5D scores were lower to those of the sex- and age-matched general population before surgery (both, *P* < 0.01), yet were similar 6 months after TAMIS.

**Table 3:** General quality of life scores

	Baseline (preoperative)	6 months after SPTS	Population
	*N* = 24	*N* = 24	*N* = 24
EQ-VAS	77 (12)	83* (14)	84 (7)
EQ-5D	82 (11)	88 (10)	89 (6)

Data are mean scores with standard deviation in parentheses. EQ-VAS score equates to QOL from a patient perspective, EQ-5D score equates to QOL from a social perspective. The population group was sex- and age- matched to the analysed patients and derived from a community-based sample of healthy individuals without co-morbidity

* *P* = 0.03 comparison with baseline

## Discussion

This is the first study focusing not only on anorectal functioning, but also on QOL following TAMIS, which makes this study unique. In this study, TAMIS proved to be a safe technique. Overall, anorectal functioning was not compromised, although in a small subset of patients FISI increased, depicting a deterioration in functioning. To the best of our knowledge, there is only one other paper describing the impact of TAMIS on anorectal functioning. In the recent study by Schiphorst et al. [15], preoperative FISI scores were higher than in the current study (mean 21 vs 10). The only obvious differences between both studies seem to be median age (median 79 vs 59 years) and median tumour size (18 vs 6 cm^2^), and this may attribute to the difference in preoperative FISI scores. Following TAMIS, in their study in 88 % of patients, continence improved, whereas in only two patients, functioning deteriorated at 6-month follow-up. In our study, in 79 % of patients, anorectal functioning improved, and in five patients, it decreased. As these numbers are limited, conclusions have to be mitigated, but in our series, deterioration occurred mainly in more distal located and larger tumours. To confirm whether these are the real contributing factors, further studies have to be awaited.

Regarding QOL, a significant improve in FIQL was observed in the subscale “coping behaviour”. Tumour size and distance from the dentate line had no effect on these FIQL scores. We also observed a better general QOL score (EQ-VAS) after TAMIS (*P* = 0.03). We can only speculate on this improvement. However, besides removal of the tumour which may have led to incontinence-like symptoms, it seems reasonable a rejoice phenomenon plays a role. Finally, social QOL (EQ-5D) improved 6 months after TAMIS, and at that time point, was comparable to the general population.

Although our study population is small, it is a very homogenous group, including only patients with adenoma or a low-risk T1 carcinoma. Only patients who solely underwent TAMIS, using only one system, were included. Hereby, the possible influence of other systems or anal retractors on functional outcome is eliminated.

How are our results compared to studies following TEM? In earlier studies, we already showed TEM has no detrimental effect on anorectal functioning [7]. A recent study by Allaix showed TEM to be safe even after long-term follow-up. Our study shows TAMIS can compete with TEM as it comes to anorectal functioning and QOL [16]. However, long-term results have to be awaited. Also, as TEM has proven safe with respect to local recurrence rates in RA and T1 rectal carcinomas, TAMIS should produce equivalent results on these aspects, before it can be embraced safely.

In conclusion, TAMIS seems to be a safe procedure without compromising anorectal functioning and improves QOL in most patients. Nonetheless, more data, especially on long-term outcome and long-term functional results, will be required before concluding it is equal to TEM, the current gold standard procedure.
